# The spatial organization of non-homologous end joining: From bridging to end joining

**DOI:** 10.1016/j.dnarep.2014.02.010

**Published:** 2014-05

**Authors:** Takashi Ochi, Qian Wu, Tom L. Blundell

**Affiliations:** Department of Biochemistry, University of Cambridge, 80 Tennis Court Road, Cambridge CB2 1GA, UK

**Keywords:** Non-homologous end joining, DNA-PKcs, Artemis, DNA ligase IV, XRCC4, XLF, Cernunnus, LIG4 syndrome

## Abstract

Non-homologous end joining (NHEJ) repairs DNA double-strand breaks generated by DNA damage and also those occurring in V(D)J recombination in immunoglobulin and T cell receptor production in the immune system. In NHEJ DNA-PKcs assembles with Ku heterodimer on the DNA ends at double-strand breaks, in order to bring the broken ends together and to assemble other proteins, including DNA ligase IV (LigIV), required for DNA repair. Here we focus on structural aspects of the interactions of LigIV with XRCC4, XLF, Artemis and DNA involved in the bridging and end-joining steps of NHEJ. We begin with a discussion of the role of XLF, which interacts with Ku and forms a hetero-filament with XRCC4; this likely forms a scaffold bridging the DNA ends. We then review the well-defined interaction of XRCC4 with LigIV, and discuss the possibility of this complex interrupting the filament formation, so positioning the ligase at the correct positions close to the broken ends. We also describe the interactions of LigIV with Artemis, the nuclease that prepares the ends for ligation and also interacts with DNA-PK. Lastly we review the likely affects of Mendelian mutations on these multiprotein assemblies and their impacts on the form of inherited disease.

## Highlights

• Structural analyses of NHEJ suggest mechanisms of DNA double-strand break repair.
• Complexes of Artemis with LigIV and DNA-PK define spatiotemporal relationships.
• Disease-causing mutations in Artemis, LigIV and XLF are explained by 3D structure.

## 1 Introduction

Non-homologous end joining (NHEJ) is an evolutionarily conserved repair system for DNA double-strand breaks (DSBs) [1]. NHEJ not only repairs DNA ends generated by DNA damage but also joins those created by V(D)J recombination and class switch recombination, which are joined in a strictly regulated way in order to maintain the gene integrity for immunoglobulin and T cell receptors in the immune system. NHEJ involves two pathways: classical NHEJ (referred to as NHEJ in this review) and alternative end joining (AEJ). NHEJ and AEJ use different proteins and AEJ requires micro-homology of DNA ends [2–6]. In this review, we focus on structural aspects of NHEJ.

**Fig. 1:**
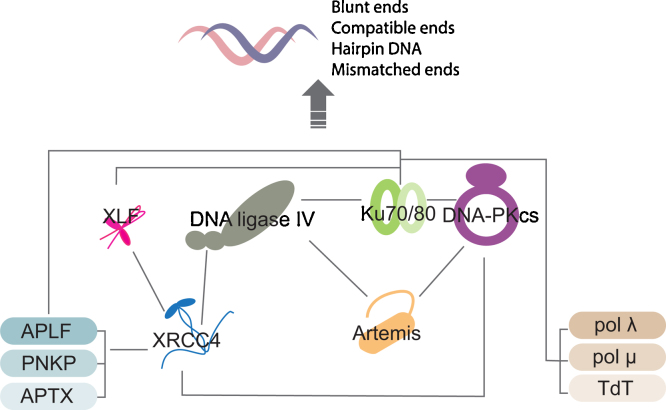
Schematic representation of the NHEJ network. Grey lines indicate protein–protein interactions [64,80,88–90,161–170].

The core components of NHEJ are the Ku70/80 heterodimer (Ku), DNA-dependent protein kinase catalytic subunit (DNA-PKcs), XRCC4, DNA ligase IV (LigIV) and XLF/Cernunnus (XLF). Ku and DNA-PKcs together with DNA form the DNA-PK complex, and XRCC4, LigIV and XLF form the NHEJ ligase complex. In addition, NHEJ requires Artemis, DNA polymerase μ and λ (pol μ and pol λ, respectively), terminal dinucletidyltrasferase (TdT), polynucleotide kinase-phosphatase (PNKP), aprataxin (APTX) and aprataxin-PNKP-like factor (APLF) [1]. 53BP1 and RIF1 play important roles in responding to DSBs in the early stages of NHEJ [7–12]. Thus, the DNA-PK and NHEJ ligase complexes share many common partners, for example, XLF, Ku and DNA itself, and contribute at DNA ends to much larger multicomponent assemblies, which vary over space and time depending on the type of DSB (Fig. 1).

## 2 DNA-PK complex

Most work on understanding the D NA-PK sub-complex has been focused on DNA-PKcs, the catalytic subunit, which belongs to the phosphoinositide 3-kinase (PI3K)-related protein kinase (PIKK) family. Several groups have analyzed the structure of DNA-PKcs using single particle reconstruction from cryo-electron microscopy [13–18]. Although these models differ quite radically between themselves, general features such as head/crown containing the kinase structure and a large circular base can be seen in all.

**Fig. 2:**
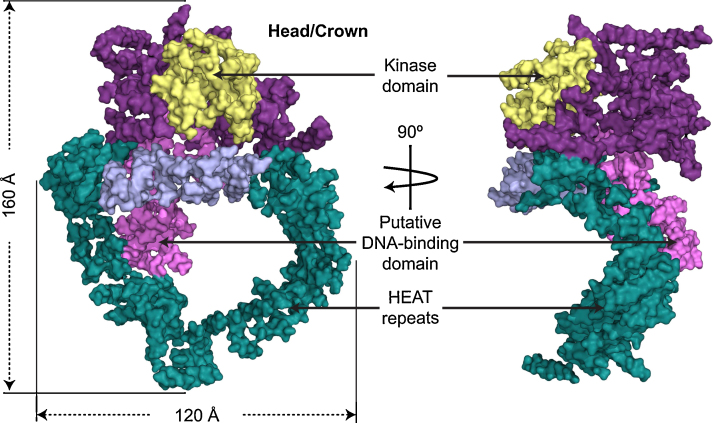
Molecular surface of the DNA-PKcs structure viewed perpendicular to the ring structure (left panel) and in the plane of the ring structure (right panel). The color code of the molecule is as follows: the ring structure is green; the forehead that is part of the ring structure is light purple; the putative DNA binding domain is pink; the larger C-terminal part is magenta, and the kinase domain is yellow (Adapted from ESRF Highlights, Newsletter, and Management Reports 2010 by BL Sibanda, D Chirgadze & TL Blundell).

Sibanda et al. (2010) produced crystals and have reported the structure of DNA-PKcs in complex with the C-terminal domain of Ku80 at 6.6 Å resolution [19]. In the electron density helical regions were clearly visible indicating that most of the structure was dominated by HEAT or other related α-helical repeats. However, the electron density for the chains linking these helices was missing in most cases, making the polypeptide path difficult to follow; the exception was the kinase domain where prior knowledge of the fold assisted the interpretation. Nevertheless, in the N-terminal region helices are clearly identified in HEAT repeats and folded into a circular structure, resembling a cradle when viewed from the side (see Fig. 2). The C-terminal region, which forms the head/crown of the molecule, is also predominantly α-helical. It contains the protein kinase domain, which is involved in phosphorylation of other proteins as well as autophosphorylation and is well positioned for access to substrates (Fig. 2). Recently Pavletich and colleagues have solved the structure of the mammalian target of rapamycin (mTOR), a further phosphoinositide 3-kinase-related protein kinase, which controls cell growth in response to nutrients and growth factors [20]. The arrangement of helices corresponds to that conservatively reported in the structure of DNA-PKcs. Ongoing X-ray structural work (BL Sibanda, D Chirgadze and TL Blundell, unpublished) at 4.3 Å resolution has defined the positions of all the equivalent helices and suggests a number of putative interaction regions shared with mTOR.

The poor resolution of the analysis and the fact that the Ku80 C-terminal domain also consists of α-helical HEAT repeats makes it difficult to locate this domain in the electron density with any certainty but it likely resides within the large N-terminal circular structure. This domain is a good candidate for DNA binding, and a putative DNA-binding sub-domain was proposed in the cryo-EM structure reported earlier [17]. Indeed the evolution of a large head domain conserved in other PI3K-related protein kinases together with a large ring structure allows DNA-PKcs to function both as an enzyme involved in DNA damage signaling and as a platform for DNA, Ku and other proteins engaged in the repair of broken DNA.

## 3 End bridging

XLF, a key protein in NHEJ, was discovered independently through yeast two-hybrid screening for XRCC4 interactors and investigations of a group of patients with growth retardation, microcephaly and immunodeficiency characterized by a profound T + B lymphocytopenia [21–23]. Down-regulation of XLF in cells causes an increase of radiosensitivity, sensitivity towards anticancer drugs, DSB repair defects and prolonged phosphorylation of histone H2AX [22]. Cells from patients carrying mutations in the *XLF* gene have impaired ability to respond to replication stress [24]. XLF is less abundant in cells compared to XRCC4 and LigIV [22]. XLF, like XRCC4, does not have enzymatic function itself, but rather performs its role in NHEJ as a scaffold protein to stabilize LigIV/XRCC4 at broken DNA ends. It enhances the LigIV/XRCC4 end-joining process specifically through LigIV readenylation following ligation [25–28]. How exactly XLF improves LigIV function and whether XLF is involved in early synapsis of NHEJ are central questions for investigation.

Structural studies of XLF and the XRCC4/XLF complex provide powerful starting points for an investigation of the functional mechanism of XLF. Despite the low sequence identity, the crystal structures of XLF and XRCC4 demonstrate that the two proteins are homologous homodimers comprising globular head domains and C-terminal helices that form coiled-coil tail structures [29–32]. The head domains form seven-stranded antiparallel β-sheets sandwiching a helix-turn-helix (HTH) motif between β4 and β5, but XLF contains an extra helix in the N-terminal region. Whereas the tail structure of XRCC4 comprises an elongated coiled-coil, the equivalent extended helix α4 of XLF is followed by further helices, α5 and α6; these fold back around the coiled-coil formed by α4 so that the C-termini come close to the α1 helices of the head domains.

**Fig. 3:**
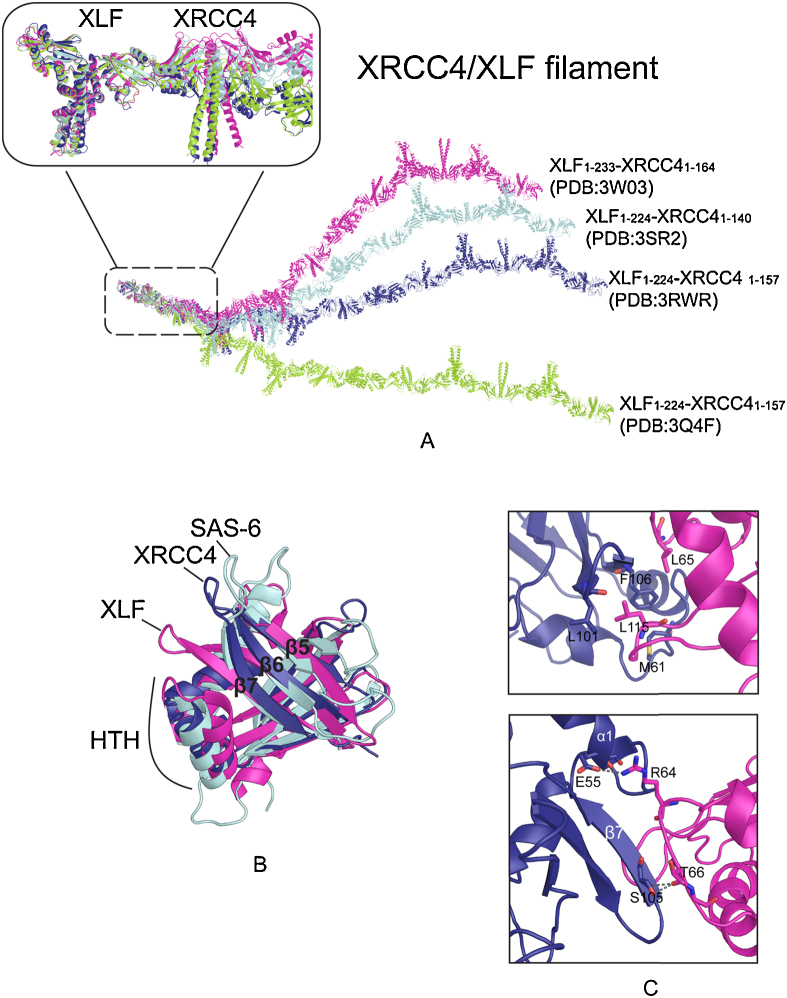
The structure of the XRCC4/XLF complex. (A) XRCC4/XLF structures solved in four different groups [34–36,38]. One turn of XRCC4/XLF filament, which contains 6 copies of each XRCC4 and XLF molecules, is generated for comparison. Superimposition of the first XLF dimer molecules demonstrates varying curvatures of the filaments. (B) Superimposition of the head domains from XLF, XRCC4 and SAS-6. β6-7 and HTH are closer together in XLF than in XRCC4 and SAS-6. The PDB codes for structures here are 1IK9 (XRCC4), 2QM4 (XLF) and 2Y3V (SAS-6) [39,40,51]. (C) The protein–protein interface of XRCC4/XLF, located in the head domain of each protein. The hydrophobic interface is shown in the top panel, while the bottom panel shows the polar interaction (indicated by grey dashed line). XLF is colored in red pink and XRCC4 is in deep purple. The XRCC4/XLF structure used is from [45].

Mutagenesis studies have demonstrated the interactions between XRCC4 and XLF are through head domains of each protein, and key interactions are conserved, exposed and located in HTH motif and β6–β7 structure for both proteins [32,33]. These key residues are symmetrically related by the dyads of the XRCC4 and XLF homodimer head domains, suggesting that XRCC4/XLF might form higher order polymers. Crystal structures, SAXS (small angle X-ray scattering) and nanospray mass spectrometry of XRCC4/XLF (both of which are C-terminal truncated) confirm this prediction [34–38]. Four structures solved in different laboratories show similar alternating XRCC4/XLF helical polymers with left-handed six-fold screw axes (Fig. 3A) [34–36,38]. The binding of the two proteins generates a tilt angle between the pseudo dyads relating the head domains and coiled-coil tail structures. The four XRCC4/XLF structures differ in the angles of rotation between the helical-tail structures of XRCC4 and XLF dimers, and this in turn leads to differences of curvature and sizes of the central cylindrical cavities when XRCC4/XLF forms higher order polymers [34–36,38] (Fig. 3A). The effects of a small twist between two head domains, with the interaction anchor regions still in touch, are amplified through the long tail structures, demonstrating that XRCC4/XLF filaments are flexible and elastic (Fig. 3C).

XLF has longer structures in the HTH loop and β6-7 strands than in XRCC4, and the distances between the HTH and β6-β7 are less for XLF than XRCC4 (Fig. 3B). Crystal structures show that the core XRCC4/XLF interface is formed mainly through hydrophobic interaction. The tips of these loops (α2-3 and β6-7) are in close proximity and form a hydrophobic patch in XLF. Key residue L115 in β6-β7 inserts into the hydrophobic pocket of XRCC4 (formed by α1-2 and β6-7) created by residues M61, L101 and F106 (Fig. 3C Top). XLF L65 (in α2-3 loop) aligns next to the XRCC4 hydrophobic pocket. In addition, XRCC4/XLF is further stabilized by polar interactions. Residues from XLF α2-3 loop R64 and T66 interact with XRCC4 E55 (in α1) and S105 (in β7), respectively (Fig. 3C Bottom). Although the interactions between XRCC4 and XLF protomers in the fibres are mediated through head domains, the C-terminal structures of XRCC4 and XLF impact positively on the strength of the interaction [36,38].

The identification of a rather small and flexible interaction region between XRCC4 and XLF should allow small molecules or peptides to be designed to disrupt the XRCC4/XLF interaction. Over-expression of XRCC4 mutants, which cannot bind to XLF, increase the radiosensitivity of wild-type CHO cells [39]. Thus, small molecules that inhibit XLF and XRCC4 interaction might prove beneficial to cancer patients for radiochemotherapy treatment. Interestingly, crystal structures of the N-terminal regions of the centriole protein SAS-6 have revealed a protein fold similar to those of XLF and XRCC4 [40–42]. Alignment of the head domains of XLF, XRCC4 and SAS-6 shows the same general folding, but a greater structural similarity between XRCC4 and SAS-6 in relative positioning of β6-7 loop and HTH structure (Fig. 3B). Indeed a higher order SAS-6 complex is formed through equivalent head-to-head interactions as seen in the XRCC4/XLF complex and coiled-coil tails of the SAS-6 dimers extend outwards towards the assemblies of microtubules.

DNA interactions with individual XRCC4 and XLF molecules are not strong and large pieces of DNA are required for stable protein–DNA interactions [27,43]. Andres and co-workers [45] have shown that XLF K293 and XRCC4 E170 and R192 are key residues for individual protein interaction with DNA. The XLF C-terminal is crucial for the interaction of the XRCC4/XLF with DNA. Thus, only full length XRCC4/XLF can mediate the DNA-bridging effect. Addition of LigIV BRCT domain or use of truncated XLF and XRCC4 proteins disables the DNA-bridging property. On the basis of these observations, two types of interaction are implicated in mediating DNA bridging: filament–DNA and filament–filament. Disruption of either leads to failure of the DNA-bridging process. The LigIV BRCT domain-binding site on XRCC4 overlaps with the probable region of XRCC4 tetramerization. Therefore this filament-filament interaction could come from the tetramerization of XRCC4 [35]. XRCC4/XLF-filament assemblies seen in both crystal structures and EM studies [34–36,38], and strong DNA binding and bridging could therefore achieved through XRCC4/XLF filament bundles containing more than one copy of the XRCC4/XLF filament. Ku and DNA-PKcs can mediate DNA synapsis [44]. XRCC4/XLF DNA bridging does not require the presence of Ku and DNA-PKcs [35]. Since Ku binds to DNA damage sites before XRCC4 and XLF, it is not known whether Ku can further improve XRCC4/XLF DNA bridging or whether they share some degree of redundant function. The C-terminal structures of XRCC4 and XLF are both targeted for phosphorylation by DNA-PKcs [45,46]. Phosphorylation of XLF residues in the unstructured C-terminal region has no effect on XLF recruitment to damaged chromatin, DNA binding and repair efficiency [47,48]. But the phosphorylation of XRCC4/XLF by DNA-PKcs can disassemble the XRCC4/XLF filament formation [39]. Therefore DNA-PKcs may be one of factors involved in XRCC4/XLF filament regulation.

The crystal structure of XRCC4 in complex with the BRCT domains of LigIV shows that the second BRCT domain of LigIV (BRCT2; residues 815-911) interacts with the coiled-coil region of XRCC4 and is positioned close to the head domain of one XRCC4 protomer [49,50]. The presence of BRCT domains bound to XRCC4 does not interfere with the formation of an individual XRCC4/XLF filament. However, in the presence of full length LigIV, filament formation is disrupted, presumably due to the presence of the LigIV catalytic domain [51], which may limit access of XLF to one side of the XRCC4 homodimer head domain and therefore reduce filament formation. This termination of the XRCC4/XLF filament formation by LigIV could be a regulatory process important for the role of the filament in the DNA double-strand break damage repair, as the XRCC4/XLF polymer would be terminated, thereby placing the ligase near a DNA end.

Alignment-based gap filling by DNA polymerase polλ and polμ in whole-cell extracts is completely dependent on XLF [52]. In XRCC4 deficient cell lines, disruption of the interaction between XLF and XRCC4 using XRCC4 mutants can restore the signal end joining, but not the coding end-joining function [39]. Therefore it is tempting to explain these functions of XLF in terms of its ability to form filaments with XRCC4, which stabilize and align the DNA ends, increasing DNA ligation efficiency.

The flexibility of the XRCC4/XLF filament opens up the possibility that it might wrap around chromatin and interact with DNA and histones. The DNA binding region of XLF would be located on the inner side of the XRCC4/XLF helical structure. DNA would wrap around the outer histones of the nucleosome as a left-handed helical structure; the XRCC4/XLF is also a left-handed helical filament structure, although the helix pitch is much greater than that of DNA super-helical packing in the nucleosome. However, it could stabilize DNA strands after nucleosome disassembly and damaged DNA is exposed for ligation. Live cell imaging techniques have identified the immediate recruitment of XLF to laser-induced DSBs with only Ku protein bound *in vivo*, and the presence of XRCC4 can stabilize XLF-DNA interaction through slowing of the highly dynamic exchange rate between bound and free XLF and DNA [53]. Protein interaction assays have confirmed the interaction between the core structure of Ku and the extreme C-terminal of XLF only in the presence of DNA while the presence of Ku abolished the DNA-length dependency of the XLF–DNA association [53]. It is also possible to accommodate Ku70/80 heterodimer within the helical fibre of XRCC4/XLF [44].

In addition to the proteins bound within the central cavity of the XRCC4/XLF helical structure, there may be other NHEJ proteins assembled on the helical tail structures of XLF and XRCC4, which are pointing outwards; this would be analogous to the assembly of proteins on the coiled-coil C-terminal regions in SAS-6. While LigIV can bind to the XRCC4 coiled-coil tail, further proteins can also interact with the C-terminal extension of XRCC4, for example PNKP [54,55]. The folded-back loop sequence between XLF α4 and α5 is evolutionarily conserved. Site-directed mutagenesis studies of XLF at L174, R178 and L179, which are all located in this evolutionarily conserved hinge region, reduces the stimulation of the DNA end-ligation activity without affecting the association with XRCC4 or DNA [32]. This XLF conserved region of unknown function may bind to other, as-yet unidentified NHEJ proteins.

Recent studies in a mouse model have shown that XLF functionally overlaps with ataxia telangiectasia-mutated protein (ATM) and XLF/ATM double-deficiency severely impairs T- and B-cell development by impairing V(D)J recombination [56]. A possible explanation is that XLF influences processes such as DNA end tethering and protecting, which are also mediated by ATM and H2AX [56]. Therefore, the function of XRCC4/XLF may not only be restricted to the final DNA-end ligation step, it could assemble in early DNA synapsis right after Ku is recruited to the DNA damaged ends. The XRCC4/XLF helical filament may act as a dynamic and regulated “reaction shell”, which stabilizes chromatin near IR foci, and gathers Ku70/80 and DNA-PKcs together for efficient NHEJ function.

## 4 End processing

End processing has been structurally well studied with and without DNA. There are excellent reviews on structural studies of the X family DNA polymerase and PNKP [57,58]. Recent crystallographic studies of APTX have provided insights into how AMP is removed from 5′-adenylated DNA [59,60]. Here we concentrate on structural aspects of Artemis, mutation of which can cause radio-sensitive severe-combined immune deficiency (RS-SCID) [61]. Excellent reviews on biological and biochemical aspects of Artemis can be found elsewhere [62,63]. Artemis, a nuclease belonging to the metallo-β-lactamase superfamily [61,64], acquires endonuclease activity by forming a complex with DNA-PKcs, which is essential for the hairpin opening in V(D)J recombination [64]. Artemis itself has been associated with a 5′-to-3′ exonuclease activity [64] but a recent study suggests that this may arise from other exonucleases co-purified from expressed cells [65]. For instance, a homologue of Artemis, RNase J, carries both endo- and 5′-to-3′ exonuclease activities [66,67] and loses both activities upon mutation of key residues in its catalytic core [68]. However, mutations of conserved residues in Artemis impair only its endonuclease function [69]. As was suggested by the authors, Artemis might have sites that are responsible for the exonuclease activity.

**Fig. 4:**
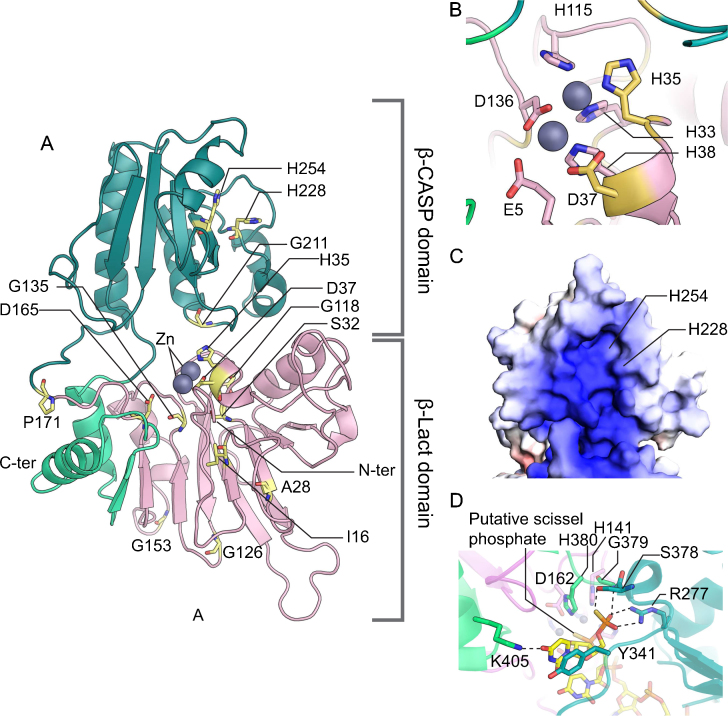
Structural model of human Artemis. (A) Overall structural model of Artemis. β-Lact and β-CASP are shown in pink and emerald green, respectively. The C-terminal fold-back of β-Lact is highlighted with light green. Positions of residues, the missense mutations of which are found in patients carrying Artemis deficiency, are shown in stick representation with yellow color. Zinc atoms are shown in sphere representation. (B) The catalytic center of Artemis. The same color scheme as (A) is used here. (C) Surface representation of β-CAPS. The positions of H228 an H254 are indicated. (D) Close-up view of the catalytic center of an archaeal RNase (PDB code: 3IEM; unpublished). Dotted lines denote hydrogen bonds.

Artemis has core metallo-β-lactamase (β-Lact) and β-CASP domains, which are conserved in nucleic acid-processing enzymes, as well as a C-terminal domain (Art-Cter), which is unique to Artemis [61,70]. Since the crystal structures of human paralogs of Artemis, CPSF-73 [71], Apollo and SNM1A (Unpublished structures; PDB codes: 3ZDK and 4B87) are available in the Protein Data Bank and the catalytic core of Artemis shares 32 and 26% sequence identity with those of Apollo and SNM1A, the structures in complex with zinc atoms can be used to build a homology model of Artemis. The model, created using Modeller [72], shows a cleft between the β-Lact and β-CASP domains and interestingly that the β-Lact domain is comprised of two polypeptides separated by the β-CASP domain (Fig. 4A).

Conserved motifs 1-4 and A-C [70] are located in the β-Lact domain and are involved in the coordination of the catalytic divalent metal ions, which are zinc in most members of the superfamily. In the catalytic core, a zinc atom is likely to be coordinated by H33, H35 and H115 (Fig. 4 B) because the equivalent zinc is present in the structure of the human paralogs. Although two zinc ions are present in the structures of CPSF-73 and Apollo, the metal ion interacting with acidic residues D37 & D136 and possibly E5 and E296 in Artemis could be magnesium and/or manganese for Artemis [64,69,73,74]. D165 is likely to form hydrogen bonds with the main-chain amides of F137 & T167 and the side chain of H319, which is a key residue for the Artemis activity [69,75], suggesting that it is structurally important. The equivalent residue of H319 in Apollo interacts with a sulfate ion and tartaric acid implying that the histidine may bind DNA. Alternatively, the divalent ion might re-arrange to be coordinated by H319 and/or D165 during nuclease catalytic activity.

The sulfate superimposes well on a phosphorothioate of an RNA analog in the structure of an archaeal RNase belonging to the β-CASP family (PDB codes: 3IEM), indicating that the sulfate mimics a scissile phosphate. The structure of *T. thermophilus* RNase J in complex with RNA shows the presence of a pocket that 5′ monophosphate binds *via* direct contacts with H243, H372, S374, G375 and H376 [76]. In the archaeal structure, the 5′ phosphorothioate forms a salt bridge with R227 and a hydrogen bond with S378 (Fig. 4D), which are equivalent to H243 and S374 of the RNase J. SNM1A and Apollo have well-conserved lysines K883 & K186 and serines S992 & S274 at the equivalent positions of H243 and S374 of the RNase J. Indeed, K186 of Apollo makes a salt bridge with co-crystallized tartaric acid. Artemis is likely to have a similar serine S317 but interestingly has a conserved tyrosine Y212 instead of lysine [70]. The difference is likely to be important for distinguishing exonuclease from endonuclease activity. In addition, Artemis is likely to have a longer loop than Apollo and SNM1A just after the first helix. Interestingly, the loop has a conserved-basic patch, which might bind the backbone of DNA.

Although the function of the β-CASP domain is not clear from the apo-structures, by analogy with the structure of archaeal (PDB code: 3IEM) and bacterial [76] orthologs of Artemis, the domain may stabilize the conformation of nucleic acids in order to enable their cleavage. Interestingly, the β-CASP domain of Artemis and its paralogs have grooves with shallow pockets, which might bind DNA (Fig. 4C).

**Fig. 5:**
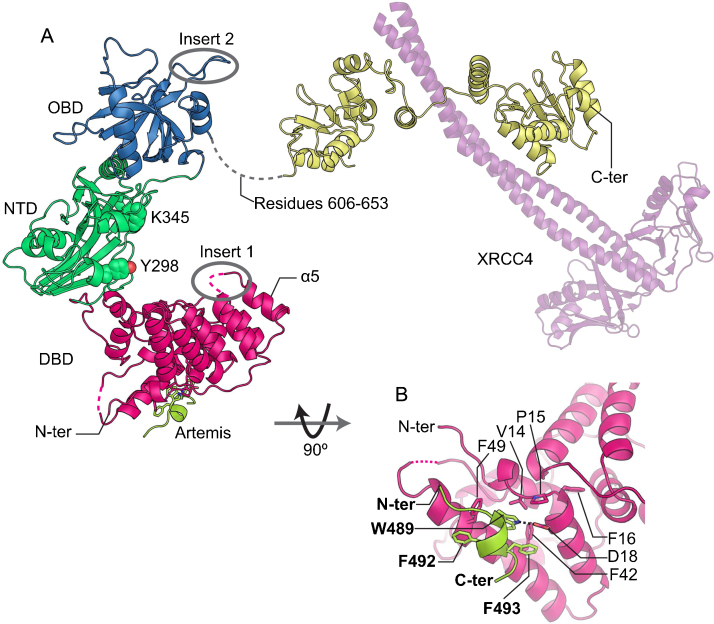
Structure of human LigIV in complex with a peptide corresponding residues 485–495 of Artemis. (A) Crystal structure of the LigIV/Artemis complex (PDB code: 3W1B; [82]) shown together with that of the LigIV/XRCC4 complex (PDB code: 3II6; [49]). DBD, NTD, OBD, BRCT domains and Artemis are shown in magenta, green, blue, yellow and lime, respectively. The XRCC4 dimer (purple) is presented in a transparent-cartoon representation. Dotted lines denote missing loops. Inset 1 and Inset 2 lies within the grey circles. Y298 and K345 are in sphere representation. (B) Details of the interaction between LigIV and Artemis. A hydrogen bond is indicated by a dotted line.

The C-terminal 300 residues (Art-Cter), which follow the core metallo-β-lactamase and β-CASP domains, are predicted to be mostly unstructured and seem to have a function in regulating Artemis endonuclease activities [73,77,78]. The details of how Art-Cter controls the endonuclease activity remain to be resolved. Importantly, the region has the DNA-PKcs and LigIV-binding motifs (residues 399–404 and 485–495, respectively) [79,80]. Recent crystallographic studies of the LigIV-binding region and LigIV complex show that Artemis and the first two helices of LigIV form a three-helical bundle mainly through hydrophobic interactions (Fig. 5B) [81,82]. Although the nature of the interaction remains to be investigated, it is clear that Artemis needs both the LigIV and DNA-PKcs interactions for an efficient coding-joint formation in V(D)J recombination [80].

In addition to these interactions, Art-Cter has PIKK phosphorylation sites concentrated after the LigIV-binding region [79,83–85]. The exact functions of the phosphorylations are not clear but they affect cell cycle [83,85] and localization [79]. If Art-Cter were highly phosphorylated after DNA damage, the net change of the region would be negative. Given that the similarity between a backbone phosphate and a phosphorylated sidechain, the phosphorylated C-terminal might interact with DNA-binding proteins including LigIV and regulate their functions. Indeed, there are examples of dynamic interactions between multi-phosphorylated peptides and globular domains [86]. Intriguingly, Artemis mutants lacking residues after T432 alter the N addition in V(D)J recombination [87], indicating that the region is important for TdT and/or pol μ functions. Since the truncated region has the LigIV-binding region, it is not clear whether LigIV or phosphorylation influence the polymerases, although both may do so because the polymerase interacts with Ku/LigIV/XRCC4 in a DNA-dependent manner [88–90].

## 5 End joining

DNA-end joining is carried out by the NHEJ ligase complex LigIV/XRCC4/XLF. This, as we have seen above, affects the activity and stability of LigIV [25,27,28,43,91–94]. XRCC4, XLF and the BRCT domains of LigIV further interact with other NHEJ proteins, but the functions of the complexes formed are not very clear. Here we focus on recent structural studies of LigIV, which have provided insights into the catalytic and other roles of the catalytic region of the protein.

LigIV, one of three human DNA ligases, is present in all eukaryotes [95,96]. LigIV has the conserved catalytic region, which is present in the ligases, followed by tandem repeats of the BRCT domain at the C-terminus, which are unique among the ligases. The characteristic fold of the catalytic region can be found in archaeal [97–100] but not in prokaryotic DNA ligases. Since the BRCT domains of LigIV were reviewed previously [101], we focus here on the catalytic region of LigIV.

The catalytic region consists of the N-terminal DNA-binding domain (DBD), a nucleotidyltransferase or adenylation domain (NTD) and an OB-fold domain (OBD) (Fig. 5A). The latter two domains have seven conserved motifs (I, III, IIIa, IV, V, Va and VI) [102,103], most of which are essential in all nucleotidyltransferases for carrying out three steps of the nucleotidyltransfer reaction: the adenylation of the catalytic lysine (step 1), the transfer of AMP to 5′ phosphate (step 2) and the joining of DNA nick (step 3)[104]. The DNA ligases undergo large conformational changes during the reaction [105]. For human and archaeal DNA ligases, there are open, closed and DNA-bound conformations of the catalytic region in the PDB, which represent neutral, step 1 and steps 2 & 3 of the reaction, respectively.

LigIV can ligate incompatible DNA ends, across gaps at DNA ends and poly-T single strands [106,107]; this, with the exception of poly-T single strands, is stimulated by Ku and XLF [106,108,109]. An unusual characteristic of LigIV is that it is difficult re-adenylate after DNA ligation [110–113], a feature that is not present in the other ligases. These observations indicate that LigIV should have unique structural features that are absent from the other human DNA ligases, LigI and LigIII. The crystal structure of the catalytic region of LigIV shows four unique features (Inserts 1 & 2, Y298 and K345 in Fig. 5A) [51,82], which are probably important for the activity of LigIV.

Insert 1 is a loop connecting α5 and α6 of DBD while Insert 2 is present within OBD. OBD in DNA ligases has the conserved motif VI, which is essential for step 1 of DNA ligation [114]. Since motif VI needs to come close to the catalytic pocket to hydrolyze ATP, OBD undergoes a large conformational change to a closed conformation [105]. However, Inserts1 and 2 stereochemically clash with OBD and DBD when LigIV has the closed conformation, a possible explanation as to why it is more difficult for LigIV to achieve the conformation and why the readenylation of LigIV is more difficult than in other human and archaeal DNA ligases. The difficulty of adenylation was observed in LigIV without XRCC4 [92] and LigIV/XRCC4 missing BRCT2 of LigIV [115]. Moreover, we have made similar observations in the dsDNA ligation assays of the catalytic region of LigIV with and without ATP [82], implying that the catalytic region of LigIV is responsible for the difficultly of readenylation.

XLF is known to stimulate readenylation of LigIV [25,93] and also interacts with LigIV *via* the first BRCT domain (BRCT1) [49]. It is unclear whether interactions of XRCC4 and XLF with LigIV take place in the context of the XRCC4/XLF filament, but they may induce conformational changes or stabilize the conformations of Inserts 1 and 2, in a way that favors the closed LigIV conformation, and stimulates adenylation.

**Fig. 6:**
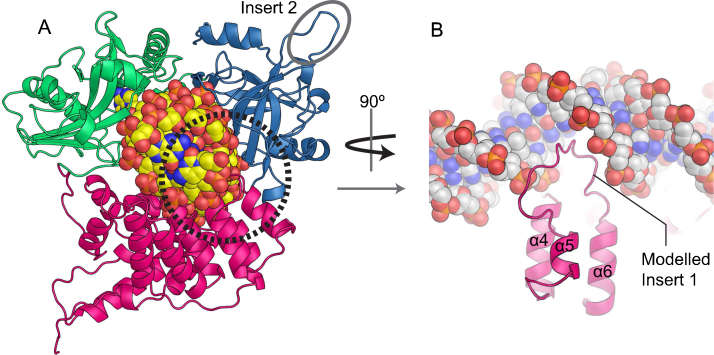
Structural model of LigIV bound nicked dsDNA. (A) Structural model of LigIV in complex with DNA. The model was built as described previously [82]. The same color scheme as in Fig. 5A is used here. (B) Model of loop between α5 & α6 fitting into the major groove of DNA. The loop was modeled using RapperTK [171].

A model of DNA-bound LigIV (Fig. 6A), based on the structures of LigI and LigIII in complex with nicked DNA [105,116], indicates that Insert 1, Y298 and K345 in NTD may be involved in the DNA-binding activity of LigIV [51,82]. Insert 1 may fit into a major grove located opposite to the DNA nick (Fig. 6B). Interestingly, the orientation of α5 with respect to α4 and α6 in DBD is different from that of the other human and archaeal DNA ligases. This might be correlated with the presence of Insert 1 and may be important for DNA ligation of the unusual substrates described above. Y298, a conserved residue in NTD of LigIV, could π stack with a base or sugar of DNA; this would be possible also in organisms where LigIV has a histidine or phenylalanine at the equivalent position.

The other residue in NTD, K345, thought to be involved in the DNA-binding activity of LigIV, is close to the 3′ OH end of the DNA nick. Most DNA ligases have phenylalanine at the equivalent position, and the structures of LigI and LigIII show that the phenylalanine π stacks with the 3′ end ribose. *E. coli* DNA ligase has arginine at the position, which is essential for the activity of the ligase [117,118]. The fact that LigIV has lysine at the position could reflect the need to detect the 3′ end flexibly. These unique features may allow LigIV to join different types of DNA ends so that DNA does not fall apart.

LigIII and LigIV but not LigI have end-joining activities towards DBSs [113]. However, although most of the DNA-binding affinity of LigI and LigIV come from DBD [105] (T.O. unpublished results), this is not true of LigIII [119]. Instead, a jack-knife model of the DNA binding of LigIII has been proposed [116,119]. Then, the question arises as to how LigIV bridges two DNA ends. XRCC4 itself forms protein filaments [37,38], which might help synapsis of DNA ends. Alternatively, LigIV might bind two fragments of DNA. Note that the linker between OBD and BRCT1 of LigIV has been shown to have affinity for DNA [120]. Moreover, the non-catalytic function of LigIV is important for autophosphorylation of DNA-PKcs implying that LigIV is an important factor for synapsis [121]. Further biochemical including structural studies of how LigIV binds DNA are required to resolve this issue.

In addition to the features related to the catalytic activity, LigIV specifically interacts with Artemis (residues 485–495) [80]. Extensive hydrophobic interactions of the helical bundle mediated by V14, F42 and F49 of LigIV and W489, F492 and F493 of Artemis make the interaction moderately stable with 4.8 μM affinity [81,82] (Fig. 5B). It is unclear how the interaction affects the activities of LigIV and/or Artemis. However, this interaction implies that LigIV can be recruited at DNA ends by Artemis forming a complex with DNA-PKcs and *vice versa*. Thus, multiple interactions among NHEJ proteins probably assemble them quickly and as stable complexes at DNA ends.

Lastly, we consider the specificity of LigIV in NHEJ. As mentioned above LigI, III and IV are likely to join two strands in a similar manner, suggesting that the catalytic regions of the ligases might replace each other with retention of function. Interestingly, mitochondrial LigIII can be replaced with LigI, LigIV and even DNA ligases from lower organisms [122,123]. It is difficult to know whether the catalytic regions of LigI and III replace that of LigIV because it is unclear whether the unique features of the catalytic region of LigIV are functionally important. However, LigI and III cannot compensate for full-length LigIV in LigIV-defective mouse [124], although LigIII can perform intermolecular ligation [113,119]. This is probably because interactions of LigIV with other macromolecules make the protein a specialized ligase for NHEJ. The interactions may be important for synapsis of correct DNA ends and/or allowing LigIV access to the ends. For instance, LigIV is specifically recruited to DNA ends by Ku [125,126] and displaces it from DNA ends [109]. Indeed, requirement of a non-catalytic function of LigIV for NHEJ has been reported [121]. In view of the fact that LigIV mutants with very weak catalytic activity cause LIG4 syndrome (see below for the details), NHEJ likely needs both catalytic and non-catalytic functions of LigIV. However, it does not eliminate the possibility that LigI and III ligate a tiny fraction of DSBs in the final step of NHEJ. Thus, it would be interesting to see whether ligases work in NHEJ in the presence of enzymatically inactive LigIV, e.g., having a mutation on K273, in order to see whether the mutation causes LIG4 syndrome or embryonic lethal. When the core components of NHEJ are missing, AEJ takes over. The zinc-finger domain of LigIII promotes DNA ligation near single-strand gaps and flaps [127], which are likely to be intermediate states of damaged DNA in AEJ, as well as intermolecular ligation [127,119]. The domain interacts with PARP-1 [128], which has been reported to play a role in AEJ [129–132]. Importantly, the zinc-finger domain is dispensable for microhomology-mediated AEJ [133]. Moreover, the same authors showed that the BRCT domain of LigIII is inessential for AEJ indicating that the ligase does not need XRCC1 for the joining. LigI also works in an alternative pathway of DSB-end joining [133,134] implying the existence of two different pathways for AEJ and a hierarchy among LigIV, III and I for the end joining [133]. Although the hierarchal mechanism remains to be elucidated, competition and crosstalk, if present among proteins involving end joining such as the ligases, Ku, PARP-1 and PAR, may decide which ligase to recruit to DSB ends. In fact, Ku directly competes with PARP-1 for DSB repair [130].

## 6 NHEJ deficiency

**Fig. 7:**
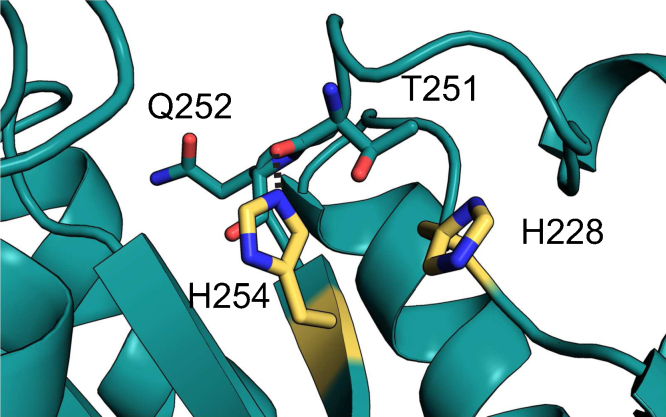
RS-SCID causing residues. (A) S32 and its surrounding residues. The same color scheme used in Fig. 5A is adopted here. (B) Residues around H228 and H254. Hydrogen bonds are indicated by dotted lines.

Mutations in *ARTEMIS*, *LIG4* and *XLF* genes are known to cause radiosensitive immune deficiency. The *ARTEMIS* gene deficiency, most frequently reported among NHEJ genes, leads to radiosensitive severe-combined immunodeficiency (RS-SCID) or Omenn syndrome [61,135]. Mutations vary from point mutations to null expression [136]. Most of the point mutants, including S32C [62], S32F [137], H35D [135], D37G [138], G118V, G135E [139] and D165V [136], are concentrated near the catalytic center of Artemis (Fig. 4A), indicating a probable loss or reduction of endonuclease function; this is confirmed by mutagenesis studies of some of the residues [69,75]. Mutations of two buried residues outside the catalytic center, I16T and A28P (Fig. 4A) found in some radiosensitive immune deficient patients [136,140], are predicted by SDM analysis [141] to result in structural instability of the β-Lact domain. It is difficult to predict the impacts of mutations G126D [142] and G153R [137], which are located in loops (Fig. 4A), without *in vitro* data and knowing the correct conformation of loop structures from the crystal structure of Artemis. P171 is in the loop connecting the β-Lact and β-CASP domains (Fig. 4A)[143]. However, a conserved proline, present at a similar position in the structures of SNM1A and Apollo, is stacked on a tyrosine in the second sub-domain of β-Lact. Since Artemis also has a tyrosine at the corresponding location, the mutation P171R may change local structure as proposed by Jeggo and colleagues [143]. Three mutations causing radiosensitive immune deficiency, G211V, H228N [136] and H254L [144], are present in the β-CASP domain (Fig. 4A). At a similar position to G211, SNM1A and Apollo have G882 and G185, which are solvent inaccessible and have positive *ϕ* torsion angles; therefore, G211V is likely to disrupt the local conformation around the residue. H254L, as indicated by a SDM analysis, destabilizes the β-CASP domain because it makes a hydrogen bond with the carbonyl oxygen of T251 (Fig. 7). Since H228 and H254 are conserved residues in the groove mentioned above in our discussion of end processing, they might have important functions apart from structural roles.

LigIV/XRCC4 is important for normal growth because the knockout of either *LIG4* or *XRCC4* gene is embryonic lethal [124,145,146], and moreover, hypomorphic mutations of LigIV or XLF in human cause rare diseases characterized conventionally by radiosensitivity, immunodeficiency, microcephaly, etc.; growth retardation and microcephaly caused by mutations in *LIG4* are classified as LIG4 syndrome [147,148]. Recent crystallographic studies of human LigIV have shed light on some point mutations, such as A3V, T9I, M249V, R278H, Q280R, H282L and G469E, which are found in LIG4 syndrome patients [149–155]. A3 and T9 are located in the flexible N-terminal region and the beginning of the first α-helix of DBD, respectively. The residues before T6 were not observed in the crystal structures of DBD [81,82], indicating that A3V is unlikely to affect structural stability or activity of LigIV. However, the increase of hydrophobicity [150] in an exposed, unstructured region caused by substitution of alanine by valine could introduce non-specific protein-protein interactions, which may interfere with the activity of LigIV. T9 stabilizes the conformation of a short α-helix connected to the following helix by a kink produced by a VPF motif. Mutation T9I may alter the local conformation, affecting the interaction with Artemis and/or DNA. The mutation increases the risk of developing severe radiation pneumonitis in some patients after radiation therapy [156]. Interestingly, A3V and T9I have protective effects on the development of multiple myeloma but cause severe clinical phenotypes when combined with R278H [157].

M249, R278, Q280, H282 and Y288, all located in NTD, are likely to be important for structural stability of the ATP-binding pocket. R278 is the only residue of this group that might interact directly with ATP [111]. R278, Q280 and H282 stabilize the conformation of the region that influences interactions between the two subdomains of NTD. Since the ATP-binding pocket lies between the subdomains, it is likely that M249V, R278H, Q280R and H282L lead to instability or conformational change in the ATP-binding pocket, resulting in a large reduction of the adenylation efficiency as reported for R278H [149]. G469, a residue in motif Va, which is important for the adenylation of LigIV [103], is completely buried and surrounded by large hydrophobic residues. Therefore, it is likely that G469E leads to disruption of the conformation of OBD.

In summary, apart from A3V and T9I, point mutations found in LIG4 syndrome mutations cause conformational changes and/or structural instabilities in LigIV.

**Fig. 8:**
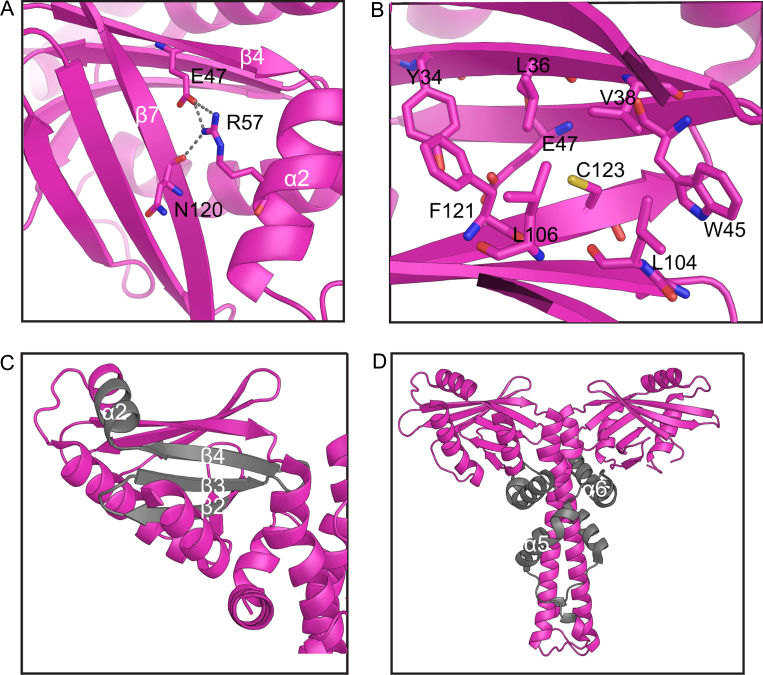
XLF mutants in Cernunnos-XLF deficiency patients. (A) R57, polar interaction is indicated by grey dashed line; (B) C123; (C) A25-R57 deletion. (D) R178X. Deletion protein sequences are shown in grey color.

Five patients, who have Cernunnos-XLF deficiency caused by mutations in *XLF* gene, have been identified so far [33]. These mutations, involving deletion between A25-R57, R57G, C123R, and R178X and resulting in truncated putative proteins, lead to severely reduced protein levels. Mutation of R57G (α2) results in loss of R57 side chain interactions with E47 (β4) and N120 (β7), which are crucial for maintaining the β-sandwich structure (Fig. 8A). C123R (β7) mutation disrupts its hydrophobic core environment created by W45 (β4), E47 (β4), Y34 (β3), L36 (β3), V38 (β3), L104 (β6), L106 (β6) and F121 (β7) (Fig. 8B). Deletion of residues between A25-R57 leads to the absence of β2-4 and half of α2 (Fig. 8C). Therefore A25-R57 deletion, R57G and C123R mutations can destabilize the XLF head domain structure and affect its function. Biochemical experiments on XLF mutant R57G also show that change of head domain conformation can result in loss of XLF ability to translocate into the nucleus properly [27]. R178X mutant is missing the fold back structure (α5, α6) and flexible C-terminal of XLF (Fig. 8D). Therefore it lacks the helix to maintain its homodimer structure, and region for DNA interaction and Ku binding.

## 7 Conclusion

Recent studies of NHEJ proteins have revealed diverse functions, which emphasize the need to reconsider the conventional NHEJ model. In this review we have focused on structural aspects of interactions of LigIV with XRCC4, XLF, Artemis and DNA, seeking to use these to inform our understanding of the spatial and temporal organization of NHEJ. We show that structural studies of LigIV/XRCC4/XLF and LigIV/Artemis complexes can shed light on their interactions at an amino-acid level, which can then be investigated *in vivo* using site-directed mutagenesis.

The DNA-double-strand-break repair process is an example of the complexity of multicomponent systems in the cell that are required to assemble and disassemble in response to signals from outside. The complexity ensures the proper colocation of components in space and time, and thus accurate and timely responses to signals outwith and within NHEJ both in immune cells and when DNA damage occurs.

Knowledge of the spatial organization and interactions between the many components of NHEJ will likely be useful for developing specific inhibitors to block the NHEJ pathway. Indeed, inhibitors of human DNA ligases including LigIV have been studied [158] and recently demonstrated to be potential drugs for cancer therapy [159]. With developing expertise in targeting protein–protein interactions [160], these complex molecular assemblies of known structure will likely become attractive targets for the development of therapeutic agents that can be used in combination with classical radio or chemotherapy.

## Conflict of interest

The authors declare that there are no conflicts of interest.
